# Artificial Intelligence and OCT Angiography in Full Thickness Macular Hole. New Developments for Personalized Medicine

**DOI:** 10.3390/diagnostics11122319

**Published:** 2021-12-08

**Authors:** Stanislao Rizzo, Alfonso Savastano, Jacopo Lenkowicz, Maria Cristina Savastano, Luca Boldrini, Daniela Bacherini, Benedetto Falsini, Vincenzo Valentini

**Affiliations:** 1 Ophthalmology Unit, Fondazione Policlinico Universitario A. Gemelli IRCCS, 00168 Rome, Italy; stanislao.rizzo@gmsil.com (S.R.); asavastano21@gmail.com (A.S.); mariacristina.savastano@gmail.com (M.C.S.); benedetto.falsini@unicatt.it (B.F.); 2 Ophthalmology Unit, Catholic University “Sacro Cuore”, 00168 Rome, Italy; luca.boldrini@policlinicogemelli.it (L.B.); vincenzo.valentini@policlinicogemelli.it (V.V.); 3 Consiglio Nazionale delle Ricerche (CNR), Istituto di Neuroscienze, 56024 Pisa, Italy; 4 Radiation Oncology Unit, Fondazione Policlinico Universitario “A. Gemelli” IRCCS, 00168 Rome, Italy; 5 Department of Neurosciences, Psychology, Drug Research and Child Health Eye Clinic, University of Florence, AOU Careggi, 50139 Florence, Italy; daniela.bacherini@gmail.com

**Keywords:** artificial intelligence, deep learning, full thickness macular hole, innovative biotechnologies, optical coherence tomography angiography, personalized medicine

## Abstract

Purpose: To evaluate the 1-year visual acuity predictive performance of an artificial intelligence (AI) based model applied to optical coherence tomography angiography (OCT-A) vascular layers scans from eyes with a full-thickness macular hole (FTMH). Methods: In this observational cross-sectional, single-center study, 35 eyes of 35 patients with FTMH were analyzed by OCT-A before and 1-year after surgery. Superficial vascular plexus (SVP) and deep vascular plexus (DVP) images were collected for the analysis. AI approach based on convolutional neural networks (CNN) was used to generate a continuous predictive variable based on both SVP and DPV. Different pre-trained CNN networks were used for feature extraction and compared for predictive accuracy. Results: Among the different tested models, the inception V3 network, applied on the combination of deep and superficial OCT-A images, showed the most significant differences between the two obtained image clusters defined in C1 and C2 (best-corrected visual acuity (BCVA) C1 = 66.67 (16.00 SD) and BCVA C2 = 49.10 (18.60 SD, *p* = 0.005)). Conclusions: The AI-based analysis of preoperative OCT-A images of eyes affected by FTMH may be a useful support system in setting up visual acuity recovery prediction. The combination of preoperative SVP and DVP images showed a significant morphological predictive performance for visual acuity recovery.

## 1. Summary Statement

Artificial intelligence-based analysis of preoperative optical coherence tomography angiography images of eyes affected by a full-thickness macular hole may be useful to support systems in setting up visual acuity recovery prediction. The combination of preoperative superficial plexus and deep plexus images showed a significant morphological predictive performance for visual acuity recovery.

## 2. Introduction

The role of advanced imaging analysis is becoming increasingly important in daily clinical practice and biomedical research due to recent advancements of radiomics and other artificial intelligence (AI)-based image analysis [1,2]. 

Clinical decision support systems are now routinely incorporated into patient evaluation workflows, often including quantitative, multimodal imaging assessments integrating several variables coming from different omics domains according to the most innovative paradigms of personalized medicine [3]. 

Ophthalmology, and more specifically retinal surgery, are exemplar subspecialties in which a remarkable use of several data sources (i.e., imaging, functional tests, electric retinal activity) is performed for the definition of comprehensive diagnosis, prognostic stratification, and follow-up strategies of patients affected by ocular diseases [4,5]. 

Owing to the application of deep learning (DL) techniques, promising AI-based models have recently been developed in ophthalmology, which incorporates different types of imaging to predict diabetic retinopathy [6], glaucoma diagnosis [7], and age-related macular degeneration [8]. 

The most common image modality used in ophthalmology is to date represented by fundus photography. Nevertheless, fundus photography has also been characterized by increasing use in recent years, including screening for blindness in diabetic eyes and for glaucoma disease [9]

Considerable interest has also been addressed to the introduction of high-resolution optical coherence tomography (OCT), not only in the ophthalmology scientific community but also for image scientists, because OCT is considered a reliable in vivo histological retinal section surrogate with up to 3.3 µm of axial resolution and significant potential for innovative AI-based investigations [10,11].

B-scan OCT images were recently evaluated in full-thickness macular holes (FTMH) for outcome prediction after surgery, paving the way towards the introduction of a more systematic application of AI in ophthalmological surgery [12]. The introduction of OCT angiography (OCT-A), which can be considered an in vivo angio-stratigraphy without dye injection, and separate analysis permitted distinguishing several different vascular layers, which brought new insights into retinal vascular structures [13,14].

Despite the promising quality of retinal vascular imaging, some images are still not immediately interpretable for the clinician due to their very recent introduction in clinical practice and the scarce available evidence [15]. As an example, different retinal diseases may influence the integrity of macular vascular layers, with the presence of a neovascular network being the easiest to detect [16,17].

Other vascular anomalies are constantly discovered with OCT-A [18].

In FTMH disease, the indication for surgery is often only supported by the reduction of visual acuity or the degree of metamorphopsia referred by the patient [19]. Although it is known that a good best-corrected visual acuity (BCVA) recovery is related to the early surgery, it does not always correspond to a suitable visual result. [20]. 

Vitreoretinal surgeons are therefore struggling to identify the most reliable morpho-functional biomarkers for surgical outcome prediction in order to identify the best therapeutic approach. The use of microperimetry recently provided useful information to determine the function-structure correlation before and after vitreoretinal surgery [21], but reliable preoperative predictors are still unavailable. 

The functional predictive information on vascular variability obtained through the qualitative analysis of OCT-A after FTMH surgery was recently described by our group showing a significant improvement of BCVA when better restoration of the vessel density (VD) was observed, especially of the deep vascular plexus (DVP) towards the superficial vascular plexus (SVP) [22]. 

Interestingly, Hu et al. observed that DL modeling of preoperative macular OCT images allows predicting postoperative FTMH status after vitrectomy and internal limiting membrane (ILM) peeling.

The aim of this study was to assess the 1-year visual acuity predictive performance of an unsupervised DL model using preoperative OCT-A scans of both, DVP and SVP.

## 3. Methods

This prospective, observational, cross-sectional study evaluated 35 eyes of 35 patients affected by FTMH evaluated with preoperative and postoperative structural OCT and OCT-A. The surgical procedure was performed by one surgeon for all the enrolled eyes. The 25-Gauge Pars Plana Vitrectomy (PPV-25G) with inner limiting membrane removal and endotamponade of sulfur hexafluoride gas (SF6) was applied. This study adheres to the Declaration of Helsinki (52nd WMA General Assembly, Edinburgh, Scotland, October 2000), and written informed consent was obtained from all patients prior to participation in the study. Institutional ethics committee approval was obtained at the Fondazione Policlinico Universitario A. Gemelli IRCCS, Università Cattolica del “Sacro Cuore”of Rome, Italy (ID number: 3680). 

**Figure 1:**
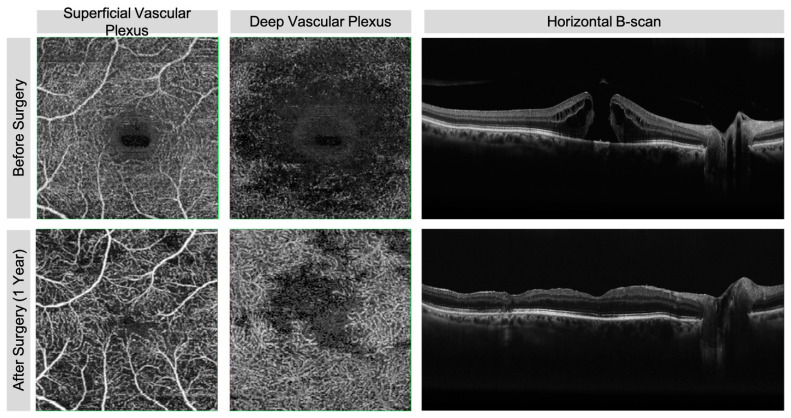
Spectral-domain structural optical coherence tomography (SD-OCT) horizontal B-scan and OCT-A before and 1 year after surgery for FTMH in eye with good visual acuity restoration. In OCT-A, the retinal vascular differences between baseline and after surgery are correspondent both in the superficial and deep vascular plexus. In B-scan at the baseline, the stromal interruption belongs to all retinal layers in the foveal region. Layer continuity is again observable 1 year after surgery including external limiting membrane (ELM) and partially the ellipsoid zone (EZ).

**Figure 2:**
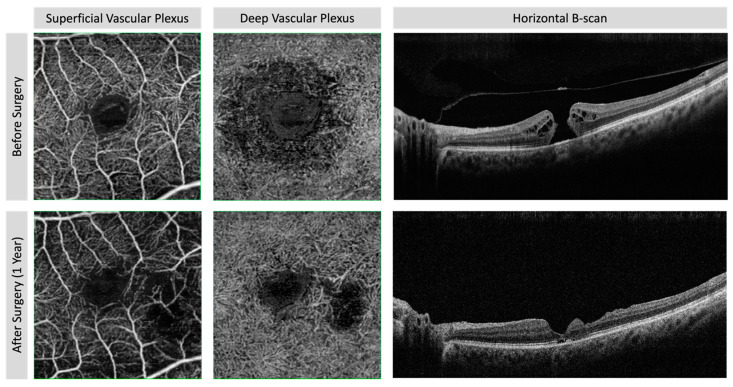
Spectral-domain structural optical coherence tomography (SD-OCT) horizontal B-scan and OCT-A before and 1-year after surgery for FTMH in eye with worsening of visual acuity. In OCT-A, the superficial and deep vascular plexuses show the vascular defect in juxta-foveal temporal region in correspondence with inner layers defect. The SD-OCT B-scan shows the ellipsoid zone defect in foveal area and the inner layer profile changes for layer defect probably after inner limiting membrane removal.

Patients were considered for this study if they were cooperative and had an FTMH detected by structural spectral-domain OCT (SD-OCT). Figure 1 shows the structural and vascular details of visual acuity good recovery after surgery. Figure 2 reports the morphological details of visual acuity increased eye after 1-year surgery.

Table 1 shows the demographic and clinical data of enrolled patients.

The acquired images had to be of adequate quality in order to define the details of vascular layers at attending ophthalmologist judgment. Possible segmentation errors were adjusted in editing modalities and propagated to all B-scans.

Exclusion criteria were media opacity and concomitant diseases such as diabetic retinopathy, vein or artery occlusion, and glaucoma. All patients underwent a baseline ophthalmic examination, including medical and ocular history, family medical history, measurement of BCVA, slit-lamp examination of the anterior and posterior segments, measurement of intraocular pressure, and dilated fundus examination. The RS-3000 Advance 2 (Nidek Co.; LTD, Gamagori, Aichi, Japan) was used to acquire SD-OCT and OCT-A. All scans were centered on the fovea based on a live scanning laser ophthalmoscopy (SLO) image. All OCT-A scans were stetted at 3 × 3 mm centered on the macula. After the complete acquisition, high-definition images of SVP and DVP were exported and used for the DL analysis.

## 4. Deep Neural Networks for Image Embedding

**Figure 3:**
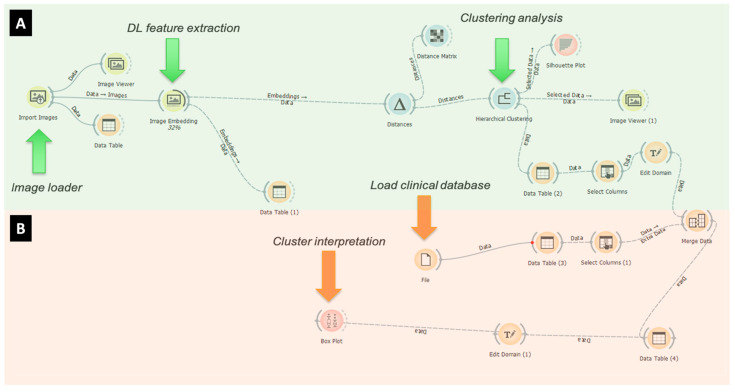
Deep learning clustering flow analysis on OCT angiography images. First steps (**A**) are represented by “image loader”, “deep learning (DL) feature extraction” and “clustering analysis” (green arrows). Second steps (**B**) are made up of “load clinical database” and “cluster interpretation” (orange arrows).

Convolutional neural networks (CNN) are a set of machine learning models that take images as input and typically produce a classification, generally represented by a continuous variable or other images as output, depending on the network architecture and the specific learning task they were trained for. In recent years, a variety of CNN architectures were developed and trained in many different computer vision scenarios, e.g., from image classification to semantic segmentation and image generation [23,24,25]. All these applications share the same underlying image processing approach, which is based on convolution kernels, a mathematical function that is repeatedly applied to the images to produce a numerical description of their morphology or texture features. The network learns how to weight the contribution of the different features according to a pre-defined loss function to maximize the accuracy of the task (Figure 3)**.**

To date, several CNN are available to advance bio-imaging studies, which can be effectively employed for image characterization tasks. 

**Figure 4:**
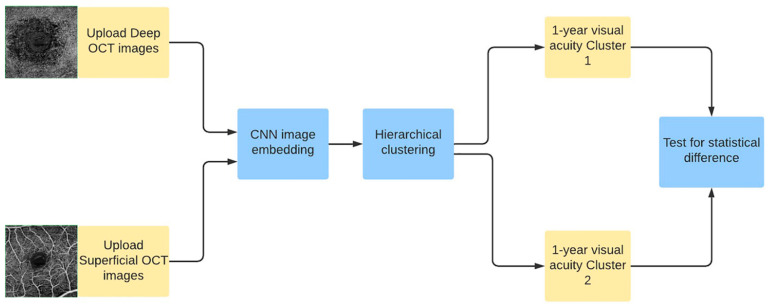
Schematic representation of the analysis workflow.

For instance, pre-trained deep CNN can be used for feature extraction from images, by removing the last classification layer of the network keeping the last dense layer, which represents the numerical categorization of the image according to the pre-trained model. As described in Figure 4, the network takes an image of a pre-defined size as input.

Layer after layer, the input image is then transformed into numeric vectors that capture the geometric characteristics of every possible sub-region of the image and patterns of pixel intensities in the same sub-regions at different levels of abstraction. Altogether, these embedded features provide a quantitative description of the image, which is completely automated, as opposed to the hand-crafted feature extraction often used in traditional radiomics analysis [26]. 

The advantage of this technique is the exploitation of highly complex models that contain billions of parameters without the need to re-train them from scratch, which would require a massive number of training images and significant computational resources. 

In fact, the model weights and coefficient are already determined and can be eventually waked for the particular task. For the scope of this analysis, several pre-trained CNN networks were used for feature extraction and compared in terms of accuracy for the specific predictive task. The following networks have been used: Inception V [27] 3, VGG-1 [28] 6, VGG-19 [29] and SqueezeNet [30].

## 5. Statistical Analysis

Sample size calculations were not necessary because our analysis is based on a computer vision perspective using classification models of radiomics images, analytical tools of machine learning, and deep learning. To mitigate the possible effect of reduced sample size, we opted for an unsupervised classification algorithm (clustering).

The feature extraction process was applied to two different classes of OCT-A images, namely superficial and deep, acquired in the preoperative settings. 

For each CNN tested, the process led to the creation of two different datasets―one for the superficial and another for the deep OCT-A images―which have a number of rows equal to the number of images included in the study, and a number of columns equal to the number of features extracted by the particular CNN. A distance matrix was then computed for the two datasets using the cosine distance metric, which was given as input to a hierarchical clustering algorithm to assign each image to one of two clusters of similar images. Once the images had been assigned to a cluster, the distribution of 1-year visual acuity score was compared between the clusters with Student’s *t*-test both for deep and superficial images. The same clustering process was then repeated on a merged dataset with half of the features obtained from deep images and half from superficial images. Again, the 1-year visual acuity score was compared between the clusters. The rationale of this approach was that a clustering on the complete superficial and deep datasets could take the best of both image types in terms of total OCT characterization. For this reason, the *t*-test *p*-values were compared to define which cluster configuration achieved the highest level of separation on the 1-year visual acuity score. The comparison was also extended to the four different CNN networks to assess whether the network architecture had an influence on the OCT-A images characterization. Statistical analysis was performed in Python version 3.7 and Orange version 3.26.

## 6. Results

Thirty-five eyes of 35 patients with FTMH were analyzed with preoperative OCT-A images. SVP and DVP high-quality images were collected and exported for the analysis. 

**Figure 5:**
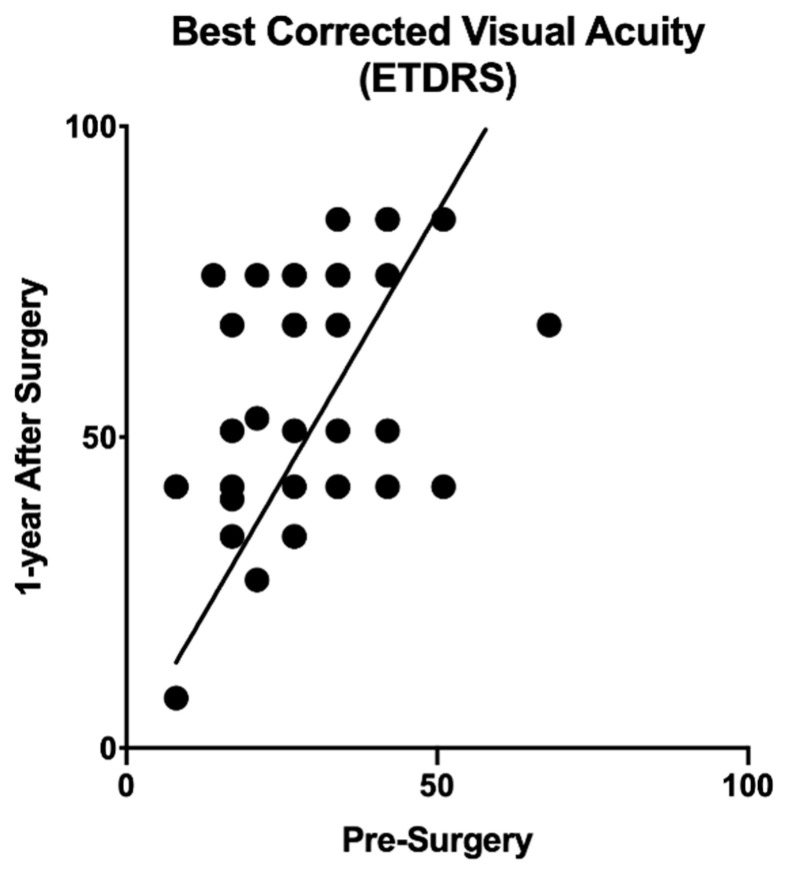
Scatterplot of visual acuity result obtained at 1-year follow up as a function of corresponding result at baseline. Solid line indicates equivalence of the values before and after surgery. Data points to the left of the equivalence line indicate improvements. Data on the right, decline. Improvements can be found in the majority of the eyes.

For a single image type, the feature extraction step produced the following number of features, depending on the network architecture: 4096 for VGG-16 and VGG-19, 2048 for Inception V3, and 1000 for the SqueezeNet. The distribution of baseline visual acuity score had a mean of 29.15 and a 13.10 standard deviation, while the 1-year BCVA score distribution had a mean of 56.63 and a standard deviation of 19.6 (Figure 5). Table with BCVA raw data at baseline and 1-year after surgery was embedded as supplemental material (Table S1) at the manuscript.

The 1-year change in visual acuity score is therefore 27.49 letters with an 18.5 letters standard deviation. 

Based on the features extracted by the four CNNs, the hierarchical clustering algorithm with a number of target clusters equal to two assigned a mean of 12.5 images to cluster 1(C1) and 22.5 images to cluster 2 (C2). 

The distribution of the 1-year visual acuity score is reported in Table 2 for the different types of CNN architectures used for feature extraction and the different type of source images.

Clustering based on SqueezeNet features from DVP assigned just one image to C1, thus its value is not included in the aforementioned table.

The most significant mean separation between the 1-year visual acuity score distributions on the two clusters defined by C1 and C2 was associated with the Inception V3 network applied on the combination of deep and superficial OCTs. 

**Figure 6:**
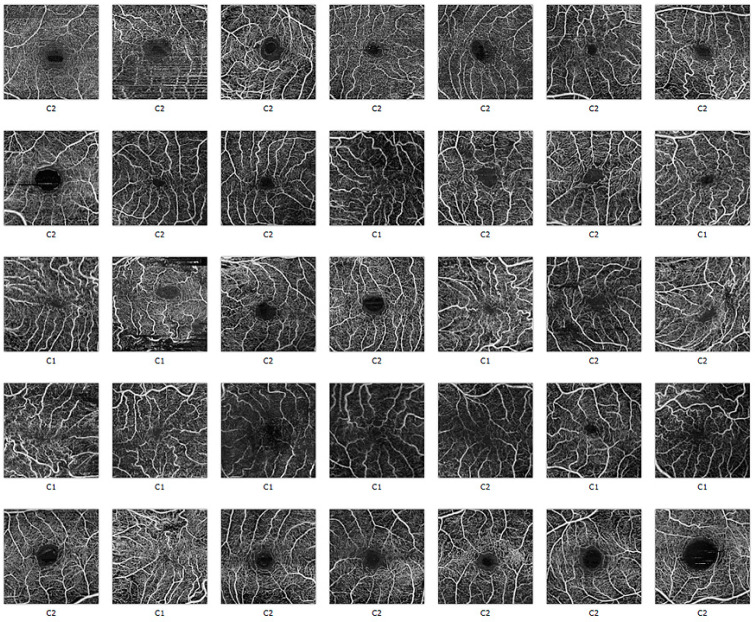
Superficial vascular plexus of OCT-A images, as assigned to Cluster 1 or 2 by the clustering algorithm based on the Inception V3 feature extraction.

**Figure 7:**
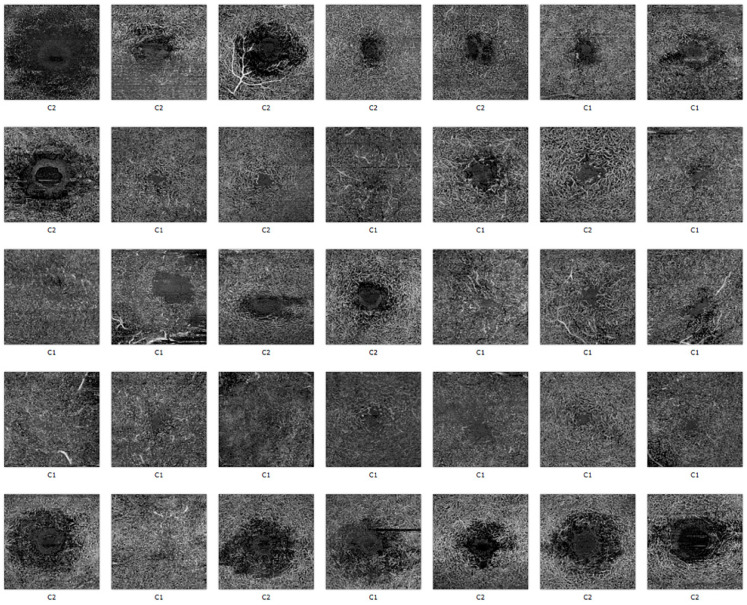
Deep vascular plexus of OCT-A images, as assigned to cluster 1 or 2 by the clustering algorithm based on the Inception V3 feature extraction.

The DL clustering assignment for the 35 superficial and deep plexus images, according to the features extracted with the Inception V3 network, are reported in Figure 6 and Figure 7.

**Figure 8:**
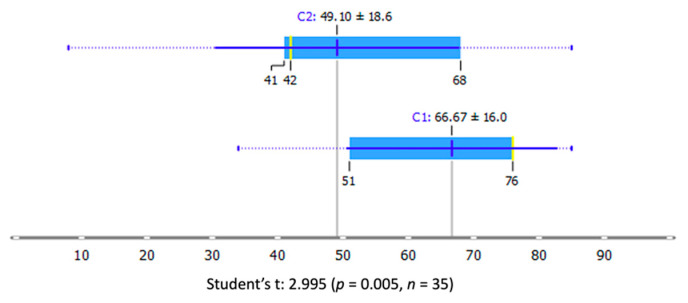
Clustering analysis from Inception V3 deep learning features based on combined superficial and deep OCT-As. The mean 1-year BVCA for C1 and C2 was 66.67 and 49.1, respectively, with a *t*-test *p*-value equal to 0.005. The yellow lines show the median values.

In this configuration, the mean of letters for C1 and C2 was 66.67 and 49.1, respectively, with a *t*-test *p*-value equal to 0.005 (Figure 8).

**Figure 9:**
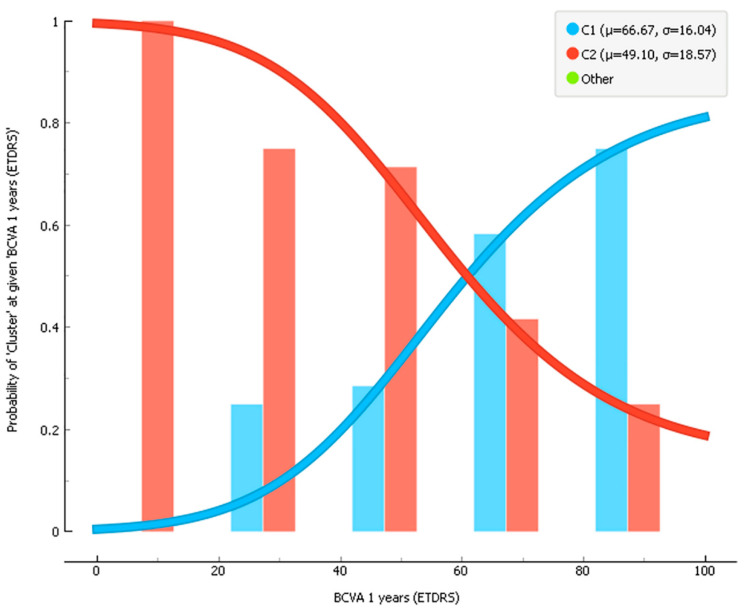
Probability belonging to a deep learning C1 or C2 given the BCVA value after 1 year. It shows that for C1 the probability increases with the increase in the BCVA value while the opposite is true for C2 since it contains all the lower BVCA values at 1 year. The green point shows that deep learning detects only the two clusters considered C1 and C2 and not others.

The probability belonging to deep learning C1 or C2, given the BCVA value after 1 year, is reported in Figure 9.

## 7. Conclusions

The annual incidence of FTMH was estimated to be 7.9 eyes per 100,000 inhabitants [30]. Small FTMH dimensions and early surgery have been both considered important prognostic factors for achieving good visual recovery following anatomically successful macular hole surgery [20]. 

Unfortunately, the achieved visual acuity restoration is often less significant than expected. One of the most crucial interests in the surgical ophthalmology research community is to set up clinical decisional support models with the aim to predict prognostic outcomes (i.e., anatomical or functional integrity). Although the possible significant variables can be multiple and originate in different data and knowledge domains (i.e., demographics, radiomics, and other omics pipelines), in most cases, the reason why some eyes improve visual acuity after surgery while others persist stable or even get worse, with apparently the same morphological image features, still remains obscure. 

Our findings suggest that the DL-based analysis of OCT-A images, acquired in the pre-operative setting may be of support to create promising decisional support systems.

To our knowledge, this is the first study including DL prediction in OCT-A images evaluation in the peer-reviewed literature (search terms in Google, Google Scholar, PubMed, and Scopus: ocular optical coherence tomography, OCT, OCTA, OCT-A, ophthalmic optical coherence tomography angiography matched with full-thickness macular hole and macular hole).

The combination of superficial and deep preoperative OCT-A images showed how the Inception V3 and SqueezeNet models were significant morphological predictors for visual acuity recovery in eyes affected by FTMH, and the different identified clusters were peculiar for visual acuity restoration after 1-year, disclosing a further potential role for AI applications on larger validation cohorts of patients. Indeed, our results reported that for C1, the probability increases with increased BCVA value while the opposite is true for C2 since it contains all the lower BVCA values at 1 year. (Figure 9)

These preliminary findings may be useful in ophthalmological clinical practice, as to date retinal specialists are inaccurate and demonstrate a significantly lower sensitivity in detecting retinal vascular details information in the case of FTMH when compared to deep learning models. 

Furthermore, the combination of SVP and DVP datasets, in terms of total features and network architecture characterization, seems to achieve a higher accuracy.

Besides the promising predictive performances, this exploratory experience still presents different pitfalls that may limit its immediate translational applications. First of all, the limited total number of used images scanned. This choice was imposed by the unavailability of patients with images of adequate quality and a sufficient follow-up length. Furthermore, the absence of an external validation cohort reduces the methodological robustness of our model. In order to address these issues, prospective validation studies are ongoing in order to confirm model robustness and replicability on more numerous patient cohorts. Further in this experience, we decided to focus on the informative value of the images only, in order to apply a more straightforward unsupervised learning experience. The integration of other variables (i.e., age, sex) will be taken into account in future studies with a specific focus on composite biomarkers. 

According to a recent study by Obata et al. [31], postoperative BCVA after FTMH treatment could be predicted via DL using preoperative OCT images. However, our unsupervised DL was trained to evaluate vascular plexus images after the promising results of a previous study [22].

In the near future, we will evaluate the potential of our algorithm also on B-scan and then compare the predictive responses of both.

Despite the aforementioned limitations, these innovative AI-based applications may successfully support ophthalmologists in functional predictive tasks and clinical decision making, representing a promising and still unexplored knowledge domain.

The application of the described model in the clinical setting of FTMH surgery represents the future perspective of this investigation. This real-world validation will provide the final proof of the model’s efficacy in predicting post-operative functional outcomes (i.e., therapeutic potential) of macular hole surgery. 

## Figures and Tables

**Table 1:** Demographic and clinical data before surgery procedure (mean ± standard deviation).

# Eyes	35
Age (years)	70.45 ± 8.24
Female/male	21/14
FTMH Size/diameter (µm)	186.28 ± 39.85
Duration of disease persistence (Months)	4.45 ± 2.5

FTMH: Full thickness macular hole.

**Table 2:** Distribution of 1-year visual acuity score in the two image clusters for the different CNN types. * *p* < 0.05; ** *p* < 0.01.

CNN Type	Image Type	1-Year Visual Acuity Mean (Standard Deviation)— Cluster 1	1-Year Visual Acuity Mean (Standard Deviation)— Cluster 2	*t*-Test *p*-Value
Inception V3	Superficial Images	59.64 (18.40)	51.52 (20.50)	0.252
	Deep Images	61.70 (17.20)	49.87 (20.50)	0.081
	Superficial + Deep Images	66.67 (16.00)	49.10 (18.60)	0.005 **
VGG-16	Superficial Images	62.29 (15.90)	52.86 (20.80)	0.139
	Deep Images	59.96 (17.6)	43.29 (21.40)	0.092
	Superficial + Deep Images	63.85 (15.40)	52.36 (20.50)	0.070
VGG-19	Superficial Images	67.80 (11.90)	52.16 (20.20)	0.008 **
	Deep Images	60.50 (18.20)	45.44 (19.20)	0.060
	Superficial + Deep Images	59.92 (14.00)	54.91 (21.70)	0.416
SqueezeNet	Superficial Images	59.03 (18.00)	45.00 (22.40)	0.196
	Deep Images	-	-	-
	Superficial + Deep Images	66.90 (13.4)	52.52 (20.10)	0.021 *

## Data Availability

The data presented in this study are available in supplementary material.

## Supplementary Materials

The following are available online at https://www.mdpi.com/2075-4418/11/12/2319/s1, Table S1. Best corrected visual acuity data at baseline and 1-year after surgery of all enrolled eyes.
