# Correction: Rizzo et al. Artificial Intelligence and OCT Angiography in Full Thickness Macular Hole. New Developments for Personalized Medicine. *Diagnostics* 2021, *11*, 2319

**DOI:** 10.3390/diagnostics12071593

**Published:** 2022-06-30

**Authors:** Stanislao Rizzo, Alfonso Savastano, Jacopo Lenkowicz, Maria Cristina Savastano, Luca Boldrini, Daniela Bacherini, Benedetto Falsini, Vincenzo Valentini

**Affiliations:** 1 Ophthalmology Unit, Fondazione Policlinico Universitario A. Gemelli IRCCS, 00168 Rome, Italy; stanislao.rizzo@gmsil.com (S.R.); asavastano21@gmail.com (A.S.); mariacristina.savastano@gmail.com (M.C.S.); benedetto.falsini@unicatt.it (B.F.); 2 Ophthalmology Unit, Catholic University “Sacro Cuore”, 00168 Rome, Italy; luca.boldrini@policlinicogemelli.it (L.B.); vincenzo.valentini@policlinicogemelli.it (V.V.); 3 Consiglio Nazionale delle Ricerche (CNR), Istituto di Neuroscienze, 56024 Pisa, Italy; 4 Radiation Oncology Unit, Fondazione Policlinico Universitario “A. Gemelli” IRCCS, 00168 Rome, Italy; 5 Department of Neurosciences, Psychology, Drug Research and Child Health Eye Clinic, University of Florence, AOU Careggi, 50139 Florence, Italy; daniela.bacherini@gmail.com

## Figure Legend

In the original publication [1], there was a mistake in the legend for Figure 8. Values for C1 and C2 were inverted. The correct legend appears below.

Figure 8 legend becomes:

Clustering analysis from Inception V3 deep learning features based on combined superficial and deep OCT-As. The mean 1-year BVCA for C1 and C2 was 66.67 and 49.1, respectively, with a *t*-test *p*-value equal to 0.005.

## Error in Table

In the original publication, there was a mistake in Table 2 as published. Values for C1 and C2 were inverted. The corrected Table 2 appears below.

**Table 2:** Distribution of 1-year visual acuity score in the two image clusters for the different CNN types. * *p* < 0.05; ** *p* < 0.01.

CNN Type	Image Type	1-Year Visual Acuity Mean (Standard Deviation)—Cluster 1	1-Year Visual Acuity Mean (Standard Deviation)—Cluster 2	*t*-Test *p*-Value
Inception V3	Superficial Images	59.64 (18.40)	51.52 (20.50)	0.252
	Deep Images	61.70 (17.20)	49.87 (20.50)	0.081
	Superficial + Deep Images	66.67 (16.00)	49.10 (18.60)	0.005 **
VGG-16	Superficial Images	62.29 (15.90)	52.86 (20.80)	0.139
	Deep Images	59.96 (17.6)	43.29 (21.40)	0.092
	Superficial + Deep Images	63.85 (15.40)	52.36 (20.50)	0.070
VGG-19	Superficial Images	67.80 (11.90)	52.16 (20.20)	0.008 **
	Deep Images	60.50 (18.20)	45.44 (19.20)	0.060
	Superficial + Deep Images	59.92 (14.00)	54.91 (21.70)	0.416
SqueezeNet	Superficial Images	59.03 (18.00)	45.00 (22.40)	0.196
	Deep Images	-	-	-
	Superficial + Deep Images	66.90 (13.4)	52.52 (20.10)	0.021 *

## Text Correction

There was an error in the original publication. Values for C1 and C2 were inverted.

A correction has been made to Abstract, sentence: best-corrected visual acuity (BCVA) C1 = 49.10 (18.60 SD) and BCVA C2 = 66.67 (16.00 SD, *p* = 0.005)

The sentence becomes:

best-corrected visual acuity (BCVA) C1 = 66.67 (16.00 SD) and BCVA C2 = 49.10 (18.60 SD, *p* = 0.005). 

A correction has been made to Results, Paragraph 9, sentence: In this configuration, the mean of letters for C1 and C2 was 49.1 and 66.67, respectively, with a *t*-test *p*-value equal to 0.005 (Figure 8)

The sentence becomes:

In this configuration, the mean of letters for C1 and C2 was 66.67 and 49.1, respectively, with a *t*-test *p*-value equal to 0.005 (Figure 8).

The authors apologize for any inconvenience caused and state that the scientific conclusions are unaffected. This correction was approved by the Academic Editor. The original publication has also been updated.
