# Supplementary material for: Artificial Intelligence and OCT Angiography in Full Thickness Macular Hole. New Developments for Personalized Medicine
Source: Diagnostics (Basel). 2021 Dec 8;11(12):2319. doi: 10.3390/diagnostics11122319 (PMC8700555; doi:10.3390/diagnostics11122319)
Supplement: Supplementary file 1 [file diagnostics-11-02319-s001.zip › diagnostics-1417961-supplementary.pdf]

Table S1. Best corrected visual acuity data at baseline and 1-year after surgery of all enrolled eyes.

Patient	BCVA (ETDRS letters)	
	Baseline	1-year after surgery
1	14	76
2	8	42
3	21	53
4	21	27
5	17	42
6	17	40
7	27	76
8	17	51
9	8	8
10	17	68
11	27	68
12	34	42
13	17	34
14	42	85
15	27	34
16	34	85
17	51	85
18	34	76
19	27	42
20	27	34
21	34	76
22	34	68
23	8	42
24	51	42
25	27	76
26	68	68
27	34	42
28	21	76
29	42	51
30	42	76
31	27	68
32	42	42
33	42	85
34	27	51
35	34	51

BCVA = Best corrected visual acuity; **ETDRS**: Early treatment diabetic retinopathy study.
